# An epidemiological surveillance study (2021–2022): detection of a high diversity of *Clostridioides difficile* isolates in one tertiary hospital in Chongqing, Southwest China

**DOI:** 10.1186/s12879-023-08666-2

**Published:** 2023-10-19

**Authors:** Yihong Cui, Chuanming Zhang, Qianying Jia, Xue Gong, Yu Tan, Xinping Hua, Wenwen Jian, Shenglin Yang, Kim Hayer, Raja Kamarudin Raja Idris, Yi Zhang, Yuan Wu, Zeng Tu

**Affiliations:** 1 Department of Pathogen Biology, College of Basic Medical Science, Chongqing Medical University, 400016 Chongqing, China; 2 Department of Laboratory Medicine, The First Affiliated Hospital of Chongqing Medical University, 400016 Chongqing, China; 3 Department of Infectious Diseases, The First Affiliated Hospital of Chongqing Medical University, 400016 Chongqing, China; 4 Leicester Medical School, University of Leicester, LE1 7RH Leicester, UK; 5 International Medical College, Chongqing Medical University, 400016 Chongqing, China; 6 State Key Laboratory of Infectious Disease Prevention and Control, National Insti for Communicable Disease Control and Prevention, Chinese Center for Disease Prevention and Control, 102206 Beijing, China

**Keywords:** *C. difficile*, Molecular epidemiology, Risk factors, Antibiotics resistance

## Abstract

**Background:**

*Clostridioides difficile* is a bacterium that causes antibiotic-associated infectious diarrhea and pseudomembranous enterocolitis. The impact of *C. difficile* infection (CDI) in China has gained significant attention in recent years. However, little epidemiological data are available from Chongqing, a city located in Southwest China. This study aimed to investigate the epidemiological pattern of CDI and explore the drug resistance of *C. difficile* isolates in Chongqing.

**Methods:**

A case-control study was conducted to investigate the clinical infection characteristics and susceptibility factors of *C. difficile.* The features of the *C. difficile* isolates were evaluated by testing for toxin genes and using multi-locus sequence typing (MLST). The susceptibility of strains to nine antibiotics was determined using agar dilution technique.

**Results:**

Out of 2084 diarrhea patients, 90 were tested positive for the isolation of toxigenic *C. difficile* strains, resulting in a CDI prevalence rate of 4.32%. Tetracycline, cephalosporins, hepatobiliary disease, and gastrointestinal disorders were identified as independent risk factors for CDI incidence. The 90 strains were classified into 21 sequence types (ST), with ST3 being the most frequent (n = 25, 27.78%), followed by ST2 (n = 10, 11.11%) and ST37 (n = 9, 10%). Three different toxin types were identified: 69 (76.67%) were A^+^B^+^CDT^−^, 12 (13.33%) were A^−^B^+^CDT^−^, and 9 (10%) were A^+^B^+^CDT^+^. Although substantial resistance to erythromycin (73.33%), moxifloxacin (62.22%), and clindamycin (82.22%), none of the isolates exhibited resistance to vancomycin, tigecycline, or metronidazole. Furthermore, different toxin types displayed varying anti-microbial characteristics.

**Conclusions:**

The strains identified in Chongqing, Southwest China, exhibited high genetic diversity. Enhance full awareness of high-risk patients with HA-CDI infection, particularly those with gastrointestinal and hepatocellular diseases, and emphasize caution in the use of tetracycline and capecitabine. These findings suggest that a potential epidemic of CDI may occur in the future, emphasizing the need for timely monitoring.

**Supplementary Information:**

The online version contains supplementary material available at 10.1186/s12879-023-08666-2.

## Background

*Clostridioides difficile* infection (CDI) is a highly prevalent diseases in healthcare settings and is the leading cause of antibiotic-related diarrhea worldwide [1]. The incidence, severity, and mortality rates of CDI have significantly increased since the emergence of hypervirulent strains of toxigenic *C. difficile* in the 21st century [2]. In the United States, CDI is responsible for an estimated 13, 000 deaths and incurs approximately $1 billion in medical expenses [3]. In Asia, CDI has been reported in 14.8% of individuals with diarrhea, with East Asia having the highest rate (19.5%) among Asian populations [4, 5]. Recently data from a meta-analysis of 14 regions of China revealed an incidence rate of 11.4% for CDI [6]. However, the epidemiology of CDI in Chongqing remains poorly understood due to limited epidemiological data.

The characteristics and genetic diversity of *C. difficile* exhibit regional variations. In Europe and North America, the most prevalent strain is ST1, whereas in Asia, ST37 is more commonly detected [7–9]. In China specifically, the predominant STs of *C. difficile* vary between northern (ST2 and ST81) and southern regions (ST54, ST3, and ST37) [6]. These regional differences highlight the importance of studying the prevalence of *C. difficile* across different geographical areas.

Antibiotic exposure is the primary risk factor for the development of CDI [10]. Moreover, the widespread use of antibiotics leads to an upsurge in drug resistance, the emergence of multi-drug-resistant strains, and a decline in the cure rate of CDI [11]. Studies have shown that *C. difficile* in China displays distinct patterns of antibiotic resistance and genotype characteristics [11–15]. While the frequency of *C. difficile* infection has been investigated in certain regions of Southwest China, such as Kunming [12, 16, 17], limited data is available regarding the epidemiological features and drug sensitivity of CDI in Chongqing. Therefore, this study aimed to investigate the prevalence, identify its risk factors, and evaluate the antibiotic sensitivity among patients in a tertiary hospital in Chongqing, China, from January 2021 to September 2022. The findings of this study have implications for prevention and management of CDI in Chongqing.

## Methods

### Study design and definitions

This case-control study was conducted from January 2021 to September 2022 at the First Affiliated Hospital of Chongqing Medical University, which is a tertiary teaching hospital located in Chongqing, Southwest China. The hospital has a total of 3200 beds and serves as the Chongqing Antibacterial Drug Resistance Monitoring Center. During the survey, diarrhea patients’ unformed stool was collected for results of toxin isolation and culture. The detection of toxin A and toxin B antigens in collected feces was carried out using the enzyme linked fluorescence assay (ELFA) (Vidas mini, Bio Merieux, France). After examining medical histories, patients who were in the hospital for more than or equal to two days and who received antibiotics prior to diarrhea were included in this case-control research. Patients who were diagnosed with HA-CDI were enrolled in the case group, whereas patients who were diagnosed with non-CDI were enrolled in the control group, based on results from toxin detection and separation cultivation Results. Except for the aforementioned patients, all other patients are classified as Community-related diarrhea. Community-related diarrhea was excluded from the case-control study due to the lack of patient information. Prior approval was obtained from the institutional review boards of Chongqing Medical University, and the requirement for informed consent was waived.

The criteria used to identify cases of diarrhea was the occurrence of three or more unformed stools within a 24-hour period. Diarrhea cases were confirmed based on positive stool test for *C. difficile* toxins, detection of toxigenic *C. difficile*, or colonoscopy/histopathologic findings indicating pseudomembranous colitis [18]. Healthcare-associated *C. difficile* infection (HA-CDI) was defined as symptoms occurring more than 48 h after admission or within 12 weeks following discharge. Cases not meeting this definition were classified as community-associated CDI (CA-CDI), which also included outpatients [19]. Any second CDI episode within 14 days of a previous positive incident was considered as a duplicate case and was excluded from the study [20]. Recurrent CDI (rCDI) referred to CDI events that occurred eight weeks after a previous incident [21–23]. Severe diarrhea was determined by the presence of bloody diarrhea, hypovolemia, leukocytosis (white blood cells > 12 × 10^9^ cells/L), hypoalbuminemia (albumin level < 20 g/L), fever (above 38 °C), or pseudomembranous colitis [24].

The following data was collected during the study: Demographics, Prior hospitalization, Disease type, Use of antibiotics, Clinical symptoms, CDI history, White blood cell count level. CDI history to determine the percentage of recurring infections. The severity of CDI was assessed by the level of the white blood cell. This is important because many patients with diarrhea had kidney damage prior to infection, making it difficult to accurately measure the severity of CDI by the creatinine level.

### *C. difficile* isolation and toxin gene detection by PCR

The analysis of stool samples was conducted using the *C. difficile* toxin A/B test kit. The positive samples were subjected to the following procedures: *(1)* Alcohol pretreatment: in short, take 1 g of the fecal sample mixed evenly with 1mL75% of alcohol and stand still for 30 min; *(2) Culturing the pretreated samples on* cefoxitin cyclomerize fructose agar (CCFA, Oxoid, UK) for three days; (3) Incubating the plates at 37 °C under anaerobic jar containing 90% N_2_ and 10% CO_2_;4). As previously noted, the strain was determined by colony and Gram staining, together with polymerase chain reaction PCR) technique to find the housekeeping gene tpi of *C. difficile* [20]; 5) Extracting DNA from *C. difficile* strain using the TIAN amp Bacteria DNA Kit, and PCR was performed to detect specific *C. difficile* toxin genes, including *tcdA*, *tcdB*, binary toxin CDT (*cdtA* and *cdtB*), and toxin regulatory genes such as *cdu2*, *cdd3*, *tcdC*, *tcdD*, and *tcdE* [25–27].

### Multi-locus sequence typing (MLST)

Multi-locus sequence typing (MLST) was carried out and evaluated as reported previously, and seven housekeeping genes (*adk*, *atpA*, *dxr*, *glyA*, *recA*, *sodA*, and *tpi*) were amplified and sequenced [28]. The result was uploaded to the *C. difficile* MLST database (https://pubmlst.org/organisms/clostridioides-difficile) and acquired the allele profile and ST. The MLST data is displayed by using the minimum spanning tree produced by BioNumerics version 7.6. In brief, the smallest generating tree represents the distribution and relationship of the MLST sequence type. The number of isolates of each related type is represented by the size of a circle. The illustration on the straight lines connecting the two circles shows different positions between them. The gray region covers the type that is less than or equal to two different spots. The colored region represents an evolutionary branch.

### Antimicrobial susceptibility testing

*C. difficile* isolates were tested for susceptibility to multiple antibiotics using agar dilution technique following the guideline set by the Clinical and Laboratory Standards Institute (CLSI) [29]. The antibiotics tested included moxifloxacin, erythromycin, rifampicin, vancomycin, tetracycline, metronidazole, clindamycin, nitazoxanide, and tigecycline. To this end, a suspension equivalent to a 0.5 McFarland standard was prepared for each isolate using nutritional broth. This suspension was then swabbed onto brucella agar supplemented with heme and vitamin K1 and 5% sheep blood. The European Committee on Antimicrobial Susceptibility Testing (EUCAST) [30] or CLSI [29] recommendations were used to evaluate the results of antibiotic susceptibility tests. Additionally, the breakpoints for rifampicin, and vancomycin were determined based on previous studies [31, 32]. Isolates that demonstrated resistance to at least three different antibiotics were classified as multi-drug resistant (MDR) [33].

### Statistical analyses

SPSS version 27 was used for data entry and analysis. The quantitative variables will be presented with mean and standard deviation if the frequency of the observations has a normal distribution; otherwise, they will be presented with median and interquartile range, while categorical variables are expressed as frequency and percentage. For the analysis of CDI-related risk factors, binary logistic regression analysis was conducted first, and then multivariate logistic analysis was conducted for the factors with *p* < 0.01. Odds ratios (*OR*), 95% confidence intervals (*CI*), and P-values were calculated to evaluate the variations across groups. The significance level was established at *p* < 0.05.

## Result

**Table 1:** The 90 CDI patients’ basic information and clinical symptoms

variable	CDI (n = 90)	Percent (%)
Gender:		
Male	51	56.67
female	39	43.33
Age:		
Age (Median, IQS)	59(23)	
0–30	6	6.67
30–65	30	33.33
> 65	54	60
Season:		
Spring (March-May)	38	42.22
Summer (June–August)	18	20
Autumn (September-November)	13	14.44
Winter (December-February)	21	23.33
Ward of hospitalization:		
Gastroenterology department	15	16.67
Department of Hematology	11	12.22
Respiratory department	5	5.56
Neurology Department	4	4.44
Rehabilitation Medicine Department	12	13.33
ICU	10	11.11
Outpatient Department	7	7.78
Other departments	26	28.89
Type of infection:		
CA-CDI	7	7.78
HA-CDI	83	92.22
Severity	7	7.78
rCDI	3	3.6
Therapeutic drugs:		
Metronidazole	8	8.89
Vancomycin	52	57.78
Tegacyclin	3	3.33
Teicoplanin	3	3.33
others	17	18.89

IQS: interquartile spacing; HA-CDI: healthcare-associated *C. difficile* infection; CA-CDI: community-acquired *C. difficile* infection; ICU: intensive care unit; Severity: A leukocyte count of greater than 15 × 10^9^/L is indicative of a serious illness; rCDI: recurrent *C. difficile* infection

### Characteristics and incidence rate of CDI in hospital

During the study period, 2084 patients with diarrhea were admitted. Among them, 85 patients had community-related diarrhea, and 239 patients with hospital-related diarrhea were identified as AAD, accounting for 11.96% (239/1999). Ninety of the overall diarrhea patients were diagnosed with CDI, with a prevalence rate of 4.32% (90/2084). Of those CDI patients, 83 were classified as HA-CDI cases (34.73%, 83/239) while seven were CA-CDI patients (8.24%, 7/85). As shown in Table 1, there was a slightly higher number of male patients (n = 51, 56.67%) compared to female patients (n = 39, 43.33%). The average age of CDI patients was 63 ± 13 years. The majority of CDI cases occurred in patients over 65 years old (n = 54, 60%), followed by those between 30 and 65 years old (n = 30, 33.33%). CDI cases were observed across different hospital departments, mainly from the gastroenterology department (16.67%), department of hematology (12.22%), and rehabilitation medicine department (13.33%). Most CDI patients were admitted during the spring months (March-May) (42.22%, 38/83), followed by the winter (December-February) (23.33%, 21/83). Among the CDI patients, seven individuals (7.78%) have severe symptoms. Additionally, three patients experienced recurrent CDI, although no hospital outbreaks were reported. Vancomycin was the most commonly prescribed antibiotic (62.65%; 52 of 83), with a 100% cure rate. Metronidazole was used in 8.34% (8/83) of cases, and 87.5% of those patients were cured. One case did not react to metronidazole treatment, requiring a 14-day of vancomycin, which eventually led to a successful outcome.

**Table 2:** Susceptibility factors and distribution characteristics of patients with CDI

variable	HA-CDI (n = 83)	Control (n = 139)	Univariate analysis		Multivariate analysis	
			*P*-value	*OR*	*P-*value	*OR* (95%*CI*)
Demographic data:						
Age ≥ 65 years	49	66	0.361	1.446		
Male	49	83	0.824	1.092		
**History of disease**:						
Gastrointestinal disease	52	30	< 0.001***	4.356	< 0.001***	4.838 (2.475, 9.458)
Hepatobiliary disease	49	26	0.04*	3.359	0.009**	2.492 (1.258, 4.939)
Cardiovascular disease	30	33	0.160	1.791		
Kidney disease	20	24	0.263	0.556		
Autoimmune disease	16	22	0.142	2.274		
Diabetes mellitus	18	14	0.182	2.088		
Surgical History	23	22	0.193	1.885		
Chemotherapy	24	8	0.508	1.465		
**Antibiotic use history**:						
No. of antibiotics (≥ 3)	25	28	0.491	1.778		
β-Lactams	27	22	0.239	1.795		
Quinolone	16	28	0.346	0.623		
Cephalosporin	54	40	0.007**	3.392	0.009**	2.451 (1.251,4.803)
Penicillins	21	34	0.559	0.733		
Aminoglycosides	8	14	0.213	0.438		
Neoglycopeptides	28	27	0.450	1.514		
Tetracyclines	8	1	0.024*	15.214	0.006**	22.459 (2.447,206.121)
Meropenem	25	26	0.144	1.894		
**Biological parameters**						
WBC count > 9.5 × 10^9^/L	24	12	0.005*	4.526	0.009*	0.010 (0.165, 0.808)
Hypoalbuminemia	31	35	0.132	1.861		

WBC: white blood cell; *OR*: odds ratio; *CI*: confidence interval, *: *p* < 0.05, **: *p* < 0.01, ***: *p* < 0.001. +: positive; -: negative

### Clinical characteristics and risk factors of CDI

In this study, 156 HA-AAD patients who were not infected were included in the case-control study as a control group, while a total of 83 patients with HA-CDI were included in the case group. However, 17 HA-AAD patients were left out of the case-control study due to incomplete case data. In actuality, 139 patients with HA-AAD were included in the control group. The clinical characteristics and risk factors associated with CDI were examined by conducting a cohort study involving 83 HA-CDI patients, employing a multivariate logistic regression model. As demonstrated in Table 2, gastrointestinal disease, hepatobiliary disease, cephalosporin, tetracyclines, and WBC count (> 9.5 × 10^9^/L) are highly associated with the CDI occurrence (*p* < 0.01). However, this study found that chronic kidney disease, quinolones, hypoproteinemia, radiotherapy, chemotherapy, and surgery, which have been reported as risk factors for CDI, were not closely related to the CDI (*p* > 0.05).

**Table 3:** Molecular typing and toxin types of 90 *C. difficile* isolates

Clade	MLST	PaLoc							CDT		No.of isolates
		tcdA	tcdB	tcdC	tcdD	tcdE	Cdu2	Cdd3	cdtA	cdtB	
1	ST2	+	+	+	+	+	+	+			10
	ST3	+	+	+	+	+	+	+			25
	ST8	+	+	+	+	+	+	+			2
	ST33	+	+	+	+	+	+	+			1
	ST35	+	+	+	+	+	+	+			3
	ST149	+	+	+	+	+	+	+			1
	ST42	+	+	+	+	+	+	+			8
	ST48	+	+	+	+	+	+	+			1
	ST54	+	+	+	+	+	+	+			6
	ST63	+	+	+	+	+	+	+			2
	ST82	+	+	+	+	+	+	+			2
	ST102	+	+	+	+	+	+	+			3
	ST129	+	+	+	+	+	+	+			2
	ST111	+	+	+	+	+	+	+			1
	ST278	+	+	+	+	+	+	+			1
	ST696	+	+	+	+	+	+	+			1
3	ST5	+	+	+	+	+	+	+	+	+	6
	ST221	+	+	+	+	+	+	+	+	+	1
	ST201	+	+	+	+	+	+	+	+	+	2
4	ST37		+	+	+	+	+	+			9
	ST81		+	+	+	+	+	+			3

MLST: multi-locus sequence typing; ST: sequence type; *PaloC*: Pathogenicity determining region of *C. difficile*; CDT: binary toxin; +: positive; -: negative

### Molecular epidemiology of *C. difficile*

**Fig. 1:**
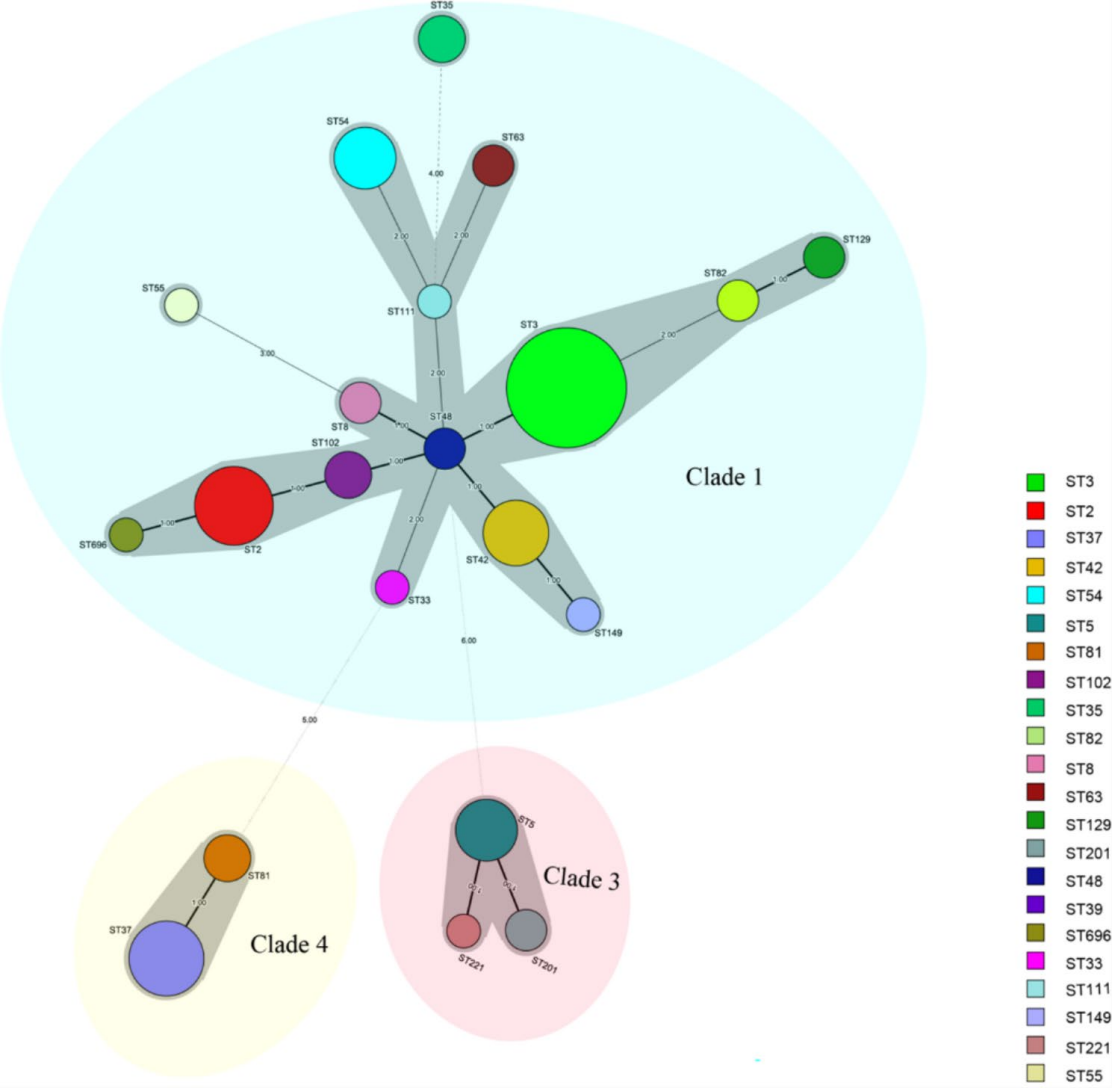
Sequence types (STs) by MLST method are displayed in the minimal spanning tree. The circle’s size represents each ST type’s separations. The number of sites between two circles appears on the straight line. Gray zones encompass all types with less than or equal to two distinct states

**Fig. 2:**
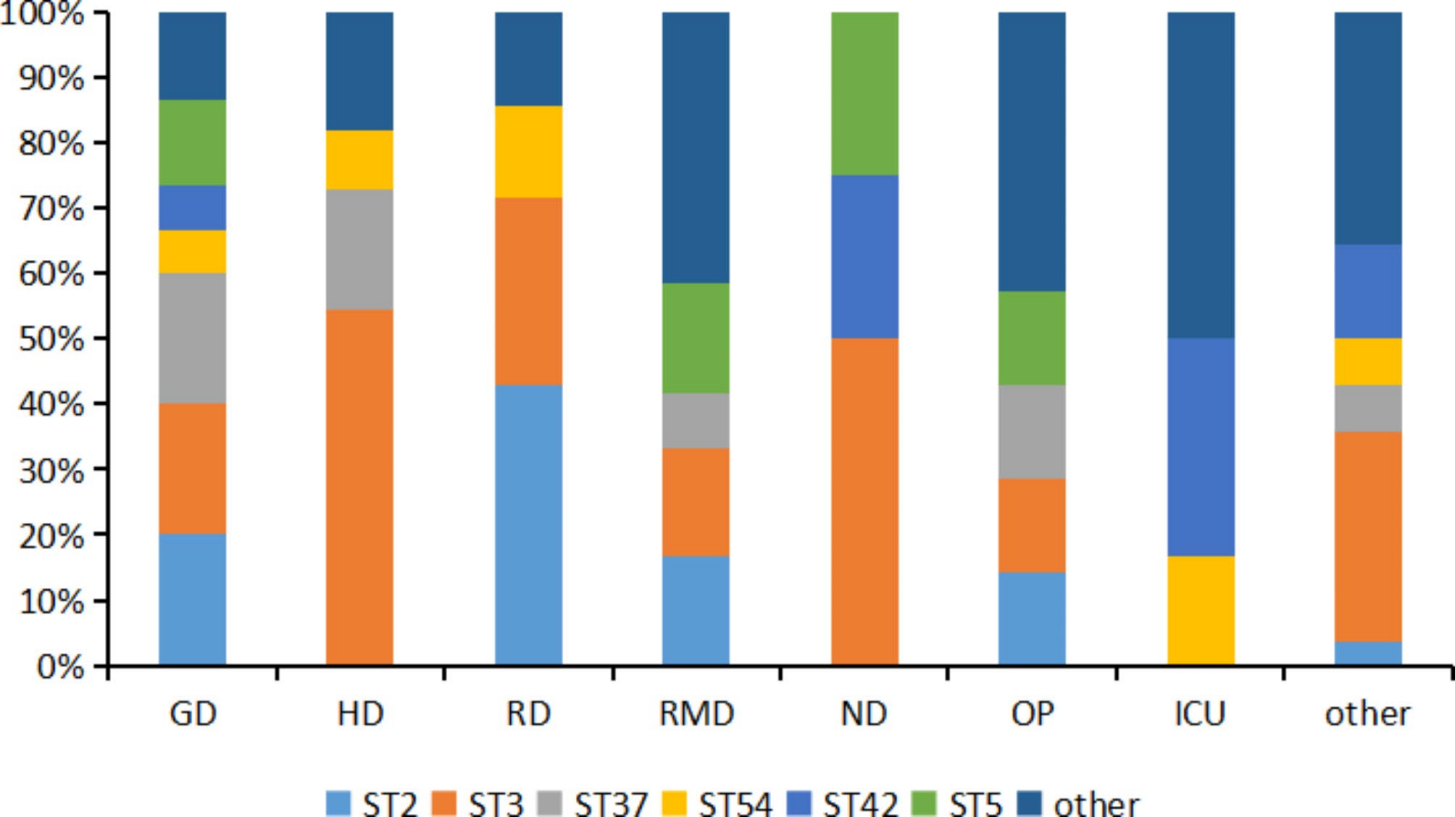
Genotype distribution by hospital departments. GD: Gastroenterology department; OP: Outpatient; HD: Hematology department; ND: Neurology department; RMD: Rehabilitation medicine department; RD: Respiratory department; ICU: Intensive care unit

**Fig. 3:**
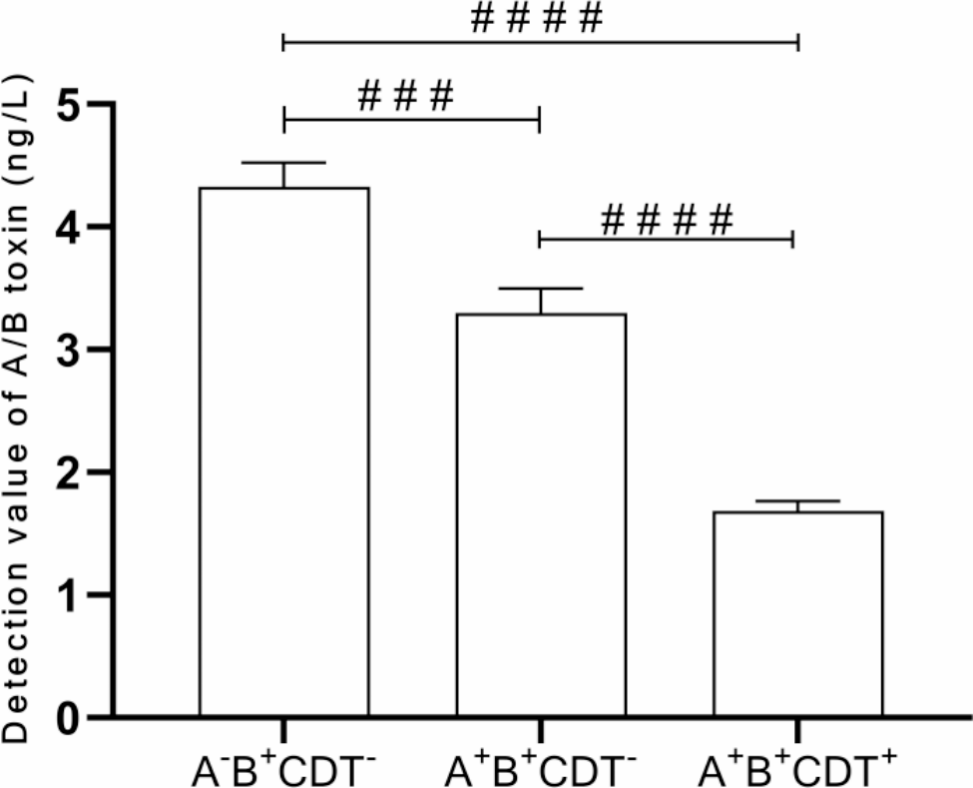
Detection value of *C. difficile* A/B toxin in stool samples from patients with three types of toxin strains. **# # #**: *p* < 0.001, **# # # #**: *p* < 0.0001

Toxins produced by *C. difficile* are strongly related to the intestinal disease. Therefore, toxin and its regulatory gene were further investigated in this study. Table 3 shows that out of a total of 90 isolates, the detected toxin gene divided them into three groups: A^+^B^+^CDT^−^ (n = 69, 76.67%), A^−^B^+^CDT^−^ (n = 12, 13.33%), and A^+^B^+^CDT^+^ (n = 9, 10%). Among 90isolated strains, 21 ST types were detected using MLST. The relationship between ST kinds was depicted in Fig. 1 by the minimum spanning tree. The most common ST type was ST3 (n = 25, 27.78%), followed by ST2 (n = 10, 11.11%), ST37 (n = 9, 10%), and ST42 (n = 8, 8.89%). These STs belong to three clade groups: clade 1 (n = 69, 76.67%), clade 3 (n = 9, 10%), and clade 4 (n = 13, 14.44%). No strains belonging to clade 2 had been found. Almost all A^−^B^+^CDT^−^ strains, except for one (ST82), belonged to clade 4 (ST37, ST81). It is worth noting that three STs, namely ST5, ST221, and ST201, were found to belong to clade 3. In terms of source of *C. difficile* in the hospital, ST3 was mainly from the hematology department, and neurology department, while ST2 was more common in the hematology department (as Fig. 2 shows). Additionally, Fig. 3 shows that CDI patients infected with A^−^B^+^CDT^−^*C. difficile* strain had higher levels of A/B toxin compared to those infected with the other two toxin strains (A^+^B^+^CDT^−^, A^+^B^+^CDT^+^).

### Detection of antimicrobial susceptibility

As shown in Table 4, the study determined the minimum inhibitory concentration (MIC) of nine antimicrobial agents for 90 isolated strains. It was found that the majority of isolates exhibited resistance to erythromycin (73.33%), moxifloxacin (62.22%), and clindamycin (82.22%). However, none of the isolates exhibited resistance to vancomycin, tigecycline, or metronidazole. Only a small percentage of isolates were resistant to rifampin (8.89%). The drug sensitivity of nitazoxanide varied between 0.125 and 4 µg/ml. Among the 90* C. difficile* isolates, 41 (45.56%) showed multiple drug resistance.

**Table 4:** Drug resistance characteristics of 90 *C. difficile* Isolates

Antibiotics	Break- point (µg/mL)	All samples (n = 90)					A^+^B^+^CDT^−^ (n = 69)				A^−^B^+^CDT^+^ (n = 12)					A^+^B^+^CDT^+^ (n = 9)			
		MIC50	MIC90	Range	Resistant rate (%)		MIC50	MIC90	Range	Resistant rate (%)	MIC50	MIC90	Range	Resistant rate (%)		MIC50	MIC90	Rang	Resistant rate (%)
Metronidazole^a^	>=32	0.25	0.5	0.125-4		0	0.125	1	0.125-4	0	0.25	0.25	0.125-1		0	0.5	0.5	0.125-2	0
Vancomycin^a^	> 2	0.031	0.125	0.031-1		0	0.063	0.125	0.031-1	0	0.063	0.125	0.031–0.125		0	0.031	0.063	0.031-1	0
Erythromycin^c^	>=8	8	32	0.25–512		73.33	4	16	0.25–256	78.26	8	32	0.25–512		83.33	4	16	1–64	33.33
Tetracycline^a^	>=16	0.5	2	0.125-8		0	0.5	4	0.125-8	10.2	0.5	0.5	0.125-4		0	2	4	0.25-4	0
Tigecycline^a^	> 0.25	0.031	0.063	0.016–0.125		0	0.031	0.063	0.031–0.125	0	0.031	0.031	0.031–0.063		0	0.016	0.031	0.016–0.063	0
Nitazoxanide^d^	——	0.5	2	0.125-4		——	0.5	1	0.125-4	——	0.5	2	0.125-4		——	1	2	0.125-4	——
Clindamycin^c^	>=8	16	64	1-512		82.22	16	16	1-128	84.05	8	8	2-512		91.67	4	8	1-128	55.56
Moxifloxacin^c^	>=8	8	16	0.25–128		62.22	8	16	0.25–128	60.87	4	8	0.5–32		50	4	8	2–32	88.89
Rifampicin^c^	>=8	0.031	0.031	0.016-64		8.89	0.063	0.063	0.031-16	5.8	0.125	16	0.031-64		25	0.016	0.063	0.016-16	11.11

^a^ MIC breakpoint is advised by the EUCAST or CLSI; ^b^ Breakpoints were proposed by a previous study; ^c^ No drug sensitivity breakpoint, report with specific values; MIC: minimum inhibitory concentration

Different antimicrobial phenotypes were observed based on toxin types. Isolates with the A^−^B^+^CDT^−^ phenotype demonstrated higher frequencies of resistance to rifampin (25%), erythromycin (83.33%), and clindamycin (91.67%), compared to those with the A^+^B^+^CDT^−^ and A^+^B^+^CDT^+^ phenotypes. Notably, isolates with A^+^B^+^CDT^+^ showed relatively high resistance rates to moxifloxacin (88.89%) compared to other drugs, although the resistance rates were still lower overall.

## Discussion

*C. difficile* is the leading cause of healthcare-associated infectious diarrhea in Europe and North America [1, 34]. But in China, particularly in the southern metropolis of Chongqing, epidemiological data about *C. difficile* are rather few [6]. The objective of the study was to evaluate the molecular epidemiology, antibiotic susceptibility, and demographic characteristics of CDI patients in Chongqing, China, between 2021 and 2022. In patients with diarrhea, the overall CDI rate in Chongqing was discovered to be 4.37% (90/2084), which was lower than that in eastern and central China [4, 29]. The study’s AAD prevalence was 11.95% (239/1999), and it was shown that HA-CDI accounted for 34.72% of AAD, consistent with other reports, demonstrating the high incidence rate of CDI in AAD patients [35].

Previous investigations have identified advanced age (≥ 65 years old), antibiotic abuse, exposure to health care environment, and various complications or diseases are the risk factors for CDI [36, 37]. Our findings, consistent with previous reports, demonstrated that advanced age (mean age: 63 years old) and antibiotics abuse were among the high-risk factors for CDI. In our study, cephalosporins (*p* = 0.009, *OR* = 2.451) and tetracycline (*p* = 0.006, *OR* = 22.459) emerged as independent significant risks for CDI. Additionally, gastrointestinal disease (*p* < 0.001, *OR* = 0.240) and hepatobiliary (*p* = 0.009, *OR* = 2.492) disease were identified as independent risk factors for CDI, highlighting the importance of intestinal barrier damage and flora disorder in CDI development [38]. Contrary to what has been shown in prior studies, our study did not find chronic renal disease to be a specific risk factor for HA-CDI. And it might be attributable to variations in patient populations and the CDI epidemic’s dynamic character [39]. Furthermore, our findings indicated that CDI could spread outside of hospitals and among younger patients, similar to some studies [40–42]. Our research hint that the risk variables for CDI change with demographics, location, and sampling period. Additionally, there is a tendency for infected populations to spread to youth and local communities. These emphasize the importance and urgency of ongoing epidemiological monitoring of CDI.

As a result of our study, we discovered a large variety of *C. difficile* isolates in Chongqing, South-west China. Based on MLST analysis, 90 separated strains were divided into 21 ST types and assigned to three evolutionary branches. The primary ST type is ST3, followed by ST2, ST37, and ST42.They differ slightly from the distribution and other parts of China, such as Beijing and Shanghai [43, 44]. This implies a regional variation in the primary types of *C. difficile* strains. However, the prevalence of certain ST types differs significantly from that observed in 2014–2016, indicating a dynamic microevolution and heterogeneity within clade 1 [39, 45], which highlights the potential evolution of *C. difficile* in Chongqing. It is worth noting that nine CDT-positive *C. difficile* belonged to clade 3, including the common ST5 seen in China and two uncommon varieties (ST201, ST221). The number and variety of CDT-positive strains have increased in 2021–2022 compared to previous studies on *C. difficile* in Chongqing from 2014 to 2016 [34]. CDT-positive strains are known to cause more severe infections, and higher mortality rates. As a result, careful observation and assessment of this strain are required [46].

Antibiotics play a crucial role in the initiation and treatment of CDI [47]. The observed trend of antimicrobial resistance in this study was consistent with previous reports [17, 37, 47], where all strains remained susceptible to first-line drugs such as metronidazole, vancomycin, and tigecycline, as indicated in Table 4. Likewise, the cure rates for metronidazole, vancomycin, and tigecycline were found to be 87.5%, 100%, and 100%, respectively, demonstrating their efficacy for treating CDI in Chongqing. However, some studies have suggested that certain *C. difficile* strains exhibit reduced susceptibility to these drugs, highlighting the need for continuous monitoring [12, 48]. Moreover, a high rate of drug resistance of the isolates against moxifloxacin, erythromycin, and clindamycin was observed, consistent with previous reports [15]. Subsequently, the MICs of nitazoxanide against *C. difficile* were investigated to assess its suitability as an alternative therapy for CDI [49]. As expected, the majority of *C. difficile* strains exhibited low MICs for nitazoxanide, suggesting its potential as a new therapeutic option for CDI. Additionally, the strain’s toxin type was found to be linked to drug resistance, with varying antibacterial characteristics among different toxin types. A^-^B^+^CDT^-^isolates demonstrated higher resistance to rifampicin (25%), erythromycin (83.33%), and clindamycin (91.67%) compared to A^-^B^+^CDT^-^ and A^-^B^+^CDT^+^ isolates. Although A^+^B^+^CDT^+^ strains had the highest resistance to moxifloxacin (88.89%), resistance was lower to other drugs. To treat and manage CDI, it may be helpful to comprehend antibiotic resistance in relation to toxin type. Furthermore, differences in drug resistance have been identified among various ST strains, highlighting the significance of genetic epidemiology research and ongoing monitoring for CDI.

Our research was limited to a single hospital in Chongqing, China, which might not accurately reflect the prevalence and variety of CDI in the general population. A multi-center investigation would enable a more thorough comprehension of CDI in Chongqing. Additionally, our study was unable to fully explore CA-CDI due to a paucity of participants and clinical data. Exploring the prevalence pattern and risk factors related to CA-CDI requires more study.

## Conclusions

In conclusion, this study presented a comprehensive survey of CDI in Chongqing, Southwest China. The overall incidence of CDI in diarrhea patients was 4.32%. Inpatients undergoing tetracycline and cephalosporin therapy and inpatients suffering from gastrointestinal disorders and hepatobiliary disease are thus at high risk for HA-CDI. Increased number and genetic diversity of *C. difficile* strains indicate the possibility of a future outbreak of hardships, and the importance of CDI continuity testing and epidemiological studies of strain molecules in Chongqing.

### Electronic supplementary material

Below is the link to the electronic supplementary material.

Supplementary Material 1

Supplementary Material 2

## Data Availability

The published paper and its supplemental information files contain the datasets created and analyzed during the current investigation. Due to the need for more research, many datasets are not currently accessible to the public but are available upon reasonable request from corresponding author.

## Abbreviations

*C*. *difficile*: *Clostridioides difficile*
*ST*: *S*equence types
CDI: *Clostridioides difficile* infection
HA-AAD: Healthcare-associated antibiotic-associated diarrhea
HA-CDI: Healthcare-associated *Clostridioides difficile* infection
CA-CDI: Community-associate *Clostridioides difficile* infection
rCDI: Recurrent *Clostridioides difficile* infection
WBC: White blood cell
CCFA: Cefoxitin cycloserine fructose agar
PCR: Polymerase chain reaction
MLST: Multi-locus sequence typing
CLSI: Clinical and Laboratory Standards Institute
EUCAST: European Committee on Antimicrobial Susceptibility Testing
MIC: Minimum inhibitory concentration
MDR: Multi-drug resistant
*OR*: *O*dds ratios
*CI*: *C*onfidence intervals
IQR: Interquartile range
ICU: Intensive care unit

## References

[1] Sandhu BK (2018). McBride SM:.Clostridioides difficile. Trends Microbiol.

[2] Warny M, Pepin J, Fang A, Killgore G, Thompson A, Brazier J, Frost E, McDonald LC (2005). Toxin production by an emerging strain of Clostridium difficile associated with outbreaks of severe disease in North America and Europe. Lancet.

[3] Guh AY, Mu Y, Winston LG, Johnston H, Olson D, Farley MM, Wilson LE, Holzbauer SM, Phipps EC, Dumyati GK (2020). Trends in U.S. Burden of Clostridioides difficile infection and outcomes. N Engl J Med.

[4] Borren NZ, Ghadermarzi S, Hutfless S, Ananthakrishnan AN (2017). The emergence of Clostridium difficile infection in Asia: a systematic review and meta-analysis of incidence and impact. PLoS ONE.

[5] Ho J, Wong SH, Doddangoudar VC, Boost MV, Tse G, Ip M (2020). Regional differences in temporal incidence of Clostridium difficile infection: a systematic review and meta-analysis. Am J Infect Control.

[6] Wen BJ, Dong N, Ouyang ZR, Qin P, Yang J, Wang WG, Qiang CX, Li ZR, Niu YN, Zhao JH (2023). Prevalence and molecular characterization of Clostridioides difficile infection in China over the past 5 years: a systematic review and meta-analysis. Int J Infect Dis.

[7] Collins DA, Sohn KM, Wu Y, Ouchi K, Ishii Y, Elliott B, Riley TV, Tateda K (2020). Clostridioides difficile infection in the Asia-Pacific region. Emerg Microbes Infect.

[8] Imwattana K, Knight DR, Kullin B, Collins DA, Putsathit P, Kiratisin P, Riley TV (2019). Clostridium difficile ribotype 017 - characterization, evolution and epidemiology of the dominant strain in Asia. Emerg Microbes Infect.

[9] Valiente E, Cairns MD, Wren BW (2014). The Clostridium difficile PCR ribotype 027 lineage: a pathogen on the move. Clin Microbiol Infect.

[10] Bignardi GE (1998). Risk factors for Clostridium difficile infection. J Hosp Infect.

[11] Tang C, Cui L, Xu Y, Xie L, Sun P, Liu C, Xia W, Liu G (2016). The incidence and drug resistance of Clostridium difficile infection in Mainland China: a systematic review and meta-analysis. Sci Rep.

[12] Yang Z, Huang Q, Qin J, Zhang X, Jian Y, Lv H, Liu Q, Li M (2020). Molecular epidemiology and risk factors of Clostridium difficile ST81 infection in a Teaching Hospital in Eastern China. Front Cell Infect Microbiol.

[13] Gu W, Li W, Jia S, Zhou Y, Yin J, Wu Y, Fu X (2022). Antibiotic resistance and genomic features of Clostridioides difficile in southwest China. PeerJ.

[14] Gu W, Wang W, Li W, Li N, Wang Y, Zhang W, Lu C, Tong P, Han Y, Sun X (2021). New ribotype Clostridioides difficile from ST11 group revealed higher pathogenic ability than RT078. Emerg Microbes Infect.

[15] Wu Y, Wang YY, Bai LL, Zhang WZ, Li GW, Lu JX (2022). A narrative review of Clostridioides difficile infection in China. Anaerobe.

[16] Liao F, Li W, Gu W, Zhang W, Liu X, Fu X, Xu W, Wu Y, Lu J (2018). A retrospective study of community-acquired Clostridium difficile infection in southwest China. Sci Rep.

[17] Jin D, Luo Y, Huang C, Cai J, Ye J, Zheng Y, Wang L, Zhao P, Liu A, Fang W (2017). Molecular Epidemiology of Clostridium difficile infection in hospitalized patients in Eastern China. J Clin Microbiol.

[18] Cohen SH, Gerding DN, Johnson S, Kelly CP, Loo VG, McDonald LC, Pepin J, Wilcox MH (2010). Clinical practice guidelines for Clostridium difficile infection in adults: 2010 update by the society for healthcare epidemiology of America (SHEA) and the infectious diseases society of America (IDSA). Infect Control Hosp Epidemiol.

[19] McDonald LC, Killgore GE, Thompson A, Owens RC, Kazakova SV, Sambol SP, Johnson S, Gerding DN (2005). An epidemic, toxin gene-variant strain of Clostridium difficile. N Engl J Med.

[20] Mora Pinzon MC, Buie R, Liou JI, Shirley DK, Evans CT, Ramanathan S, Poggensee L, Safdar N (2019). Outcomes of Community and Healthcare-onset Clostridium difficile infections. Clin Infect Dis.

[21] van Prehn J, Reigadas E, Vogelzang EH, Bouza E, Hristea A, Guery B, Krutova M, Norén T, Allerberger F, Coia JE (2021). European Society of Clinical Microbiology and Infectious Diseases: 2021 update on the treatment guidance document for Clostridioides difficile infection in adults. Clin Microbiol Infect.

[22] McDonald LC, Gerding DN, Johnson S, Bakken JS, Carroll KC, Coffin SE, Dubberke ER, Garey KW, Gould CV, Kelly C (2018). Clinical practice guidelines for Clostridium difficile infection in adults and children: 2017 update by the infectious Diseases Society of America (IDSA) and society for Healthcare Epidemiology of America (SHEA). Clin Infect Dis.

[23] Kelly CR, Fischer M, Allegretti JR, LaPlante K, Stewart DB, Limketkai BN, Stollman NH (2021). ACG clinical guidelines: Prevention, diagnosis, and treatment of Clostridioides difficile infections. Am J Gastroenterol.

[24] Alalawi M, Aljahdali S, Alharbi B, Fagih L, Fatani R, Aljuhani O (2020). Clostridium difficile infection in an academic medical center in Saudi Arabia: prevalence and risk factors. Ann Saudi Med.

[25] Braun V, Hundsberger T, Leukel P, Sauerborn M, von Eichel-Streiber C (1996). Definition of the single integration site of the pathogenicity locus in Clostridium difficile. Gene.

[26] Lemee L, Dhalluin A, Testelin S, Mattrat MA, Maillard K, Lemeland JF, Pons JL (2004). Multiplex PCR targeting tpi (triose phosphate isomerase), tcdA (toxin A), and tcdB (toxin B) genes for toxigenic culture of Clostridium difficile. J Clin Microbiol.

[27] Kato H, Kato N, Watanabe K, Iwai N, Nakamura H, Yamamoto T, Suzuki K, Kim SM, Chong Y, Wasito EB (1998). Identification of toxin A-negative, toxin B-positive Clostridium difficile by PCR. J Clin Microbiol.

[28] Eyre DW, Peto TEA, Crook DW, Walker AS, Wilcox MH. Hash-based Core Genome Multilocus sequence typing for Clostridium difficile. J Clin Microbiol 2019, 58(1). 10.1128/JCM.01037-19 PMC6935933 31666367

[29] CLSI (2017). Performance standards for antimicrobial susceptibility testing. 27th ed.CLSI supplement, M100.

[30] The European Committee on Antimicrobial Susceptibility. Testing Breakpoint tables for interpretation of MICs and zone diameters Version 100; 2010 http://wwweucastorg.

[31] Bourgault AM, Lamothe F, Loo VG, Poirier L (2006). In vitro susceptibility of Clostridium difficile clinical isolates from a multi-institutional outbreak in Southern Québec, Canada. Antimicrob Agents Chemother.

[32] Mutlu E, Wroe AJ, Sanchez-Hurtado K, Brazier JS, Poxton IR (2007). Molecular characterization and antimicrobial susceptibility patterns of Clostridium difficile strains isolated from hospitals in south-east Scotland. J Med Microbiol.

[33] Magiorakos AP, Srinivasan A, Carey RB, Carmeli Y, Falagas ME, Giske CG, Harbarth S, Hindler JF, Kahlmeter G, Olsson-Liljequist B (2012). Multidrug-resistant, extensively drug-resistant and pandrug-resistant bacteria: an international expert proposal for interim standard definitions for acquired resistance. Clin Microbiol Infect.

[34] Bassetti M, Villa G, Pecori D, Arzese A, Wilcox M (2012). Epidemiology, diagnosis and treatment of Clostridium difficile infection. Expert Rev Anti Infect Ther.

[35] Nasiri MJ, Goudarzi M, Hajikhani B, Ghazi M, Goudarzi H, Pouriran R (2018). Clostridioides (Clostridium) difficile infection in hospitalized patients with antibiotic-associated diarrhea: a systematic review and meta-analysis. Anaerobe.

[36] Bloomfield MG, Sherwin JC, Gkrania-Klotsas E (2012). Risk factors for mortality in Clostridium difficile infection in the general hospital population: a systematic review. J Hosp Infect.

[37] Banawas SS. Clostridium difficile Infections: A Global Overview of Drug Sensitivity and Resistance Mechanisms. *Biomed Res Int* 2018, 2018:8414257. 10.1155/2018/8414257 PMC5841113 29682562

[38] Abt MC, McKenney PT, Pamer EG (2016). Clostridium difficile colitis: pathogenesis and host defence. Nat Rev Microbiol.

[39] Dai W, Yang T, Yan L, Niu S, Zhang C, Sun J, Wang Z, Xia Y (2020). Characteristics of Clostridium difficile isolates and the burden of hospital-acquired Clostridium difficile infection in a tertiary teaching hospital in Chongqing, Southwest China. BMC Infect Dis.

[40] Lessa FC, Gould CV, McDonald LC (2012). Current status of Clostridium difficile infection epidemiology. Clin Infect Dis.

[41] Johnson SW, Brown SV, Priest DH (2020). Effectiveness of oral vancomycin for Prevention of Healthcare Facility-Onset Clostridioides difficile infection in targeted patients during systemic antibiotic exposure. Clin Infect Dis.

[42] Rubin ZA, Martin EM, Allyn P (2018). Primary Prevention of Clostridium difficile-Associated Diarrhea: current controversies and future tools. Curr Infect Dis Rep.

[43] Huang H, Fang H, Weintraub A, Nord CE (2009). Distinct ribotypes and rates of antimicrobial drug resistance in Clostridium difficile from Shanghai and Stockholm. Clin Microbiol Infect.

[44] Liu XS, Li WG, Zhang WZ, Wu Y, Lu JX (2018). Molecular characterization of Clostridium difficile isolates in China from 2010 to 2015. Front Microbiol.

[45] Stabler RA, Dawson LF, Valiente E, Cairns MD, Martin MJ, Donahue EH, Riley TV, Songer JG, Kuijper EJ, Dingle KE (2012). Macro and micro diversity of Clostridium difficile isolates from diverse sources and geographical locations. PLoS ONE.

[46] Stewart DB, Berg A, Hegarty J (2013). Predicting recurrence of C. difficile colitis using bacterial virulence factors: binary toxin is the key. J Gastrointest Surg.

[47] O’Grady K, Knight DR, Riley TV (2021). Antimicrobial resistance in Clostridioides difficile. Eur J Clin Microbiol Infect Dis.

[48] Spigaglia P (2016). Recent advances in the understanding of antibiotic resistance in Clostridium difficile infection. Ther Adv Infect Dis.

[49] Musher DM, Logan N, Bressler AM, Johnson DP, Rossignol JF (2009). Nitazoxanide versus vancomycin in Clostridium difficile infection: a randomized, double-blind study. Clin Infect Dis.

